# Phytotherapy in the Management of Diabetes: A Review

**DOI:** 10.3390/molecules23010105

**Published:** 2018-01-04

**Authors:** Paolo Governa, Giulia Baini, Vittoria Borgonetti, Giulia Cettolin, Daniela Giachetti, Anna Rosa Magnano, Elisabetta Miraldi, Marco Biagi

**Affiliations:** 1 Department of Physical Sciences, Earth and Environment, University of Siena, Via Laterina 8, 53100 Siena, Italy; baiocca89@gmail.com (G.B.); borgonetti@student.unisi.it (V.B.); giugiucettola@gmail.com (G.C.); daniela.giachetti@unisi.it (D.G.); annarosa.magnano@unisi.it (A.R.M.); elisabetta.miraldi@unisi.it (E.M.); biagi4@unisi.it (M.B.); 2 Italian Society of Phytotherapy, Via Laterina 8, 53100 Siena, Italy; 3 Department of Biotechnology, Chemistry and Pharmacy, University of Siena, Via Aldo Moro 2, 53100 Siena, Italy

**Keywords:** phytotherapy, hyperglycemia, diabetes, medicinal plants

## Abstract

Phytotherapy has long been a source of medicinal products and over the years there have been many attempts to use herbal medicines for the treatment of diabetes. Several medicinal plants and their preparations have been demonstrated to act at key points of glucidic metabolism. The most common mechanisms of action found include the inhibition of α-glucosidase and of AGE formation, the increase of GLUT-4 and PPARs expression and antioxidant activity. Despite the large amount of literature available, the actual clinical effectiveness of medicinal plants in controlling diabetes-related symptoms remains controversial and there is a crucial need for stronger evidence-based data. In this review, an overview of the medicinal plants, which use in the management of diabetes is supported by authoritative monographs, is provided. References to some species which are currently under increasing clinical investigation are also reported.

## 1. Introduction

In the last decade, the concept of metabolic syndrome (MS) has been extensively debated by the scientific community [1,2,3]. Despite the difficulty in establishing an exhaustive definition [4], MS is nowadays recognized as a major cardiovascular disease risk factor by the World Health Organization (WHO) and other institutions such as the International Diabetes Federation (IDF) [5,6,7]. MS can be defined as a concurrence of conditions, including obesity, hypertension, dyslipidemia and altered glycaemia [8]. MS is associated with a higher risk of type 2 diabetes and cardiovascular diseases onset [9] and involves about 25% of the world’s adult population [10], with women having a higher risk of developing MS [11].

Phytotherapy has long been a source of medicinal products and there have been many attempts to use herbal medicines for the treatment of diabetes over the years [12,13]. Furthermore, the number of scientific publications regarding herbal medicine and type 2 diabetes is continuously increasing [14].

Among the possible mechanisms of action of natural products in diabetes, such as the inhibition of α-glucosidase and α-amylase, the effects on glucose uptake and glucose transporters, the enhancement of insulin secretion and of pancreatic β-cell proliferation, the inhibition of protein tyrosine phosphatase 1B activity and the antioxidant activity have been studied in depth [15].

Despite the large amount of available literature, the real clinical effectiveness of medicinal plants in the management of diabetes is still controversial and there is a crucial need for stronger evidence-based data [16]. Indeed, despite a long folk medicine history, most of the popular species used suffer from clinical inconsistency, mainly due to the poor quality of the clinical studies [17]. Another aspect to be considered is the variability of the raw herbal materials and preparations used, which may lead to the non-reproducibility of results among different trials [18].

The aim of this review is to provide an overview of the use of medicinal plants in the management of diabetes, focusing on the species that are supported by authoritative documents such as the monographs drafted by the World Health Organization (WHO). Furthermore, an emphasis on some of the most promising species, which are attracting the interest of the scientific community, is also provided.

## 2. Medicinal Plants Used for the Management of Diabetes

Medicinal plants possessing therapeutic uses in diabetes and supported by clinical data or described in pharmacopoeias and well established documents are covered in the WHO monographs and are reported in Table 1.

### 2.1. Allium cepa L., Bulbus

*Allium cepa* L. is a perennial herb belonging to the Amaryllidaceae. The parts of the plant used are the fresh or dried bulbs, commonly known as onion, which are commercially cultivated worldwide [19]. The main chemical constituents are sulfur-containing compounds, such as l-cysteine sulfoxides, and flavonoids, such as quercetin and its glycosides [20]. *A. cepa* seems to exert its antidiabetic activity regardless of the form in which it is administered (i.e., extracts [21,22,23,24], juice [25], freeze-dried powder [26], essential oil [27]) [28].

A preliminary study evaluated the hypoglycemic effects of the oral administration of small slices of *A. cepa* (100 g/day) in type 1 and type 2 diabetic patients. Onion exhibited significant antidiabetic effects, reducing fasting blood glucose by about 89 mg/dL in type 1 diabetes patients and by 40 mg/dL in type 2 diabetes patients. A reduction of the induced hyperglycemia by 120 mg/dL in the diabetes 1 group and by 159 mg/dL in the type 2 diabetes was also observed [29].

In 2009, an in vivo study demonstrated that *A. cepa* (7% freeze-dried onion powder added into control diet) may represent an interesting anti-hyperglycaemic dietary adjunct for diabetic therapy, since it decreases serum cholesterol, triacylglycerol and LDL-cholesterol in streptozocin-induced diabetics rats, without alterations in the cholesterol and HDL-cholesterol levels [30]. Hyperglycemia causes glucose autoxidation, impaired mitochondrial bioenergetics and induces reactive oxygen species (ROS) production, leading to an impairment of intracellular pathways (i.e., JAK/STAT, JNK, p38, ERK/MAPK) and to insulin resistance [31]. Onion (400 mg/day) possesses a significant free radical-scavenging property and exerts a regulation on lipid metabolism, decreasing superoxide dismutase activity and lowering lipid hydroperoxide and lipoperoxide concentrations in diabetic rats [32].

*A. cepa* exerts its antidiabetic activity through multiple pharmacologic actions attributed to the presence of many active constituents: for example, quercetin is responsible for α-glucosidase inhibition [33] and, along with rutin, for the increase of GLUT-4 translocation, glucose uptake and insulin action [34]. Differently, l-cysteine sulfoxides and allyl propyl disulphide can act directly as free radicals scavengers. In fact, they take part in the redox process of glutathione and cysteine, and can also increase the activity of superoxide dismutase and catalase, independently or through the stimulation of insulin secretion [35,36].

### 2.2. Azadirachta indica A. Juss., Folium

*Azadirachta indica* A. Juss., also known as neem, is a deciduous tree belonging to the Meliaceae. The parts of the plant used are the dried leaves [37]. It contains characteristic compounds, such as oxidized tetranotriterpenes, known as azadirachtins [38].

An ethanolic extract (400 mg/kg) obtained from neem leaves demonstrated several effects, such as anti-lipid peroxidation, anti-hyperglycaemic and anti-hypercholesterolaemic activities as well as a reduction in serum triglyceride levels in alloxan-induced diabetic rats [39].

Two water extracts were also tested in high-fat diet-induced diabetic rats (400 mg/kg) and in normal and alloxan-induced diabetic rabbits (500 mg/kg), showing a partial prevention of the rise in blood glucose levels and a normalization of the altered levels of serum insulin, lipid profile and insulin signaling molecules as well as GLUT-4 proteins [40,41].

Chloroform extracts activities were studied in vitro and in vivo in streptozocin-induced diabetic rats (200–300 mg/kg) produced an attenuation of non-enzimatic glycation, inhibiting advanced glycation end product (AGE) formation, and alleviated oxidative stress, increasing the levels of antioxidant enzymes, glucose-6-phosphatase, hepatic glycogen content and insulin plasma levels, and decreasing glucokinase and lipid peroxidation [42,43]. The main mechanism of action of azadirachtins (e.g., azadirachtolide, azadiradione, gedunin and meliacinolin) is the inhibition of α-amylase and α-glucosidase [44,45,46].

### 2.3. Momordica charantia L., Fructus

*Momordica charantia* L. is a monoecious annual climbing vine belonging to the Cucurbitaceae. The parts of the plant used are the fresh or dried fruits, known as bitter melons [47]. The main chemical constituents are sterols, triterpenes and bioactive proteins [48].

The clinical potential of bitter melon has been examined administering capsules or tablets containing preparations from bitter melon fruits or leaves, in diabetic patients. No statistically significant improvement of blood glucose control, in terms of normalization or reduction of fasting blood glucose, reduction of glycosylated haemoglobin A1c or fructosamine, compared to placebo have been observed [49,50,51]. More recently, *M. charantia* capsules (500 mg of dried powder of the fruit pulp, containing 0.04–0.05% of charantin), administered in type 2 diabetes patients (2000 mg/day), demonstrated a significant decrease in fructosamine levels after 4 week of treatment and no side effects were observed [52].

The whole plant and/or different plant parts such as fruit pulp, seeds, and leaves have been reported to possess hypoglycemic and anti-hyperglycemic activities in several animal models: in particular, bitter melon reduced blood glucose levels and increased plasma insulin [53].

The activation of the AMP-activated protein kinase (AMPK) system and a role of the α- and γ-peroxisome proliferator-activated receptors (PPARα and PPARγ) have been hypothesized as possible mechanisms of action for *M. charantia* in diabetes treatment [54,55,56].

Cucurbitane triterpenoids from *M. charantia* displayed hypoglycemic effect, reducing blood glucose levels, moderating insulin secretion activity, increasing glucose uptake through stimulation of GLUT-4 translocation and increasing the phosphorylation of AMPK and insulin receptor substrate-1 [57,58,59,60,61].

Among bitter melon triterpenoids, 5β,19-epoxy-25-methoxy-cucurbita-6,23-diene-3β,19-diol (EMCD) and momordin have been studied in detail; it has been observed that EMCD (20 μM) can suppress the TNF-α induced expression of iNOS and nuclear translocation of NF-κB in FL83B mouse liver cells [62], while momordin is responsible for the increase of PPARδ mRNA expression at nanomolar concentrations in HepG2 human liver cells [63].

Despite the copious data from in vitro and in vivo studies, the available clinical data are often flawed by small sample size, lack of control and poor study designs. Better-designed clinical trials with sufficient sample size and statistical power will be indispensable to further confirm the efficacy of *M. charantia* as a natural treatment for diabetes mellitus [64].

### 2.4. Ocimum tenuiflorum L., Folium

*Ocimum tenuiflorum* L., commonly referred to as tulsi, is a herb or shrub up to 1 meter high belonging to the botanical family Lamiaceae. It is indigenous to India and parts of north and eastern Africa, Hainan Island and Taiwan, China and the herbal substance is represented by the fresh or dried leaves [65]. The main chemical components are tannins and essential oil (mainly composed of eugenol, methyleugenol, and α- and β-caryophyllene) [66].

In 1996, a randomized, placebo-controlled, crossover single blind trial analyzed the effects of *O. tenuiflorum* and *O. album* leaves on fasting and postprandial blood glucose and serum cholesterol levels in patients with noninsulin-dependent diabetes mellitus. A significant decrease in fasting and postprandial blood glucose levels, a similar trend in urine glucose, and a mild reduction of mean total cholesterol levels during treatment period were observed [67].

More recently, a randomized, parallel group, open label pilot study investigated the effect of tulsi extract on metabolic and biochemical parameters in 30 young overweight/obese subjects. The supplementation with *O. tenuiflorum* capsules (250 mg twice daily for 8 weeks) decreased plasma insulin and insulin resistance by 28.49% and 24.79% respectively, caused the normalization of serum lipid profile, and reduced body weight and BMI, compared to the control group (no intervention) [68].

Animal studies showed that the oral administration of *O. tenuiflorum* aqueous extracts (200 mg/kg) could delay the development of insulin resistance. Indeed, tulsi determined a considerable improvement in fasting blood glucose and in glucose tolerance and a correction of the abnormal lipid profile, through the reduction of serum total and LDL cholesterol levels. An increased activity of antioxidant enzymes (i.e., glutathione peroxidase, glutathione S-transferase, superoxide dismutase and catalase), as well as the increase of reduced glutathione levels, have been proposed as a mechanism of action for the decreased lipid peroxidation induced by tulsi [69,70,71].

Ethanol (80% *w*/*w*) extracts obtained from *O. tenuiflorum* leaves were effective in lowering blood glucose levels in normal, glucose fed hyperglycemic and streptozocin-induced diabetic rats, also potentiating the action of exogenous insulin in normal rats [72]. The glucose lowering effects was found to be mediated through its insulin secretagogue effects on ex vivo rat pancreas and in BRIN-BD11 rat clonal β-cells [73].

A reduction in the level of hepatic lipids and the reversion of the diminution of lipoprotein lipase, plasma postheparin lipolytic and lecithin cholesterol acyl transferase activities was obtained by administering 500 mg/kg of a different *O. tenuiflorum* ethanolic (95% *w*/*w*) leaves extract for 15 days in streptozocin-induced diabetic rats [74].

It has been proposed that the fixed oil extracted from *O. tenuiflorum* leaves (46.54 mg/kg/day for three weeks) may be responsible for the free radical scavenging activity, for the decrease in plasma glucose and for the increase in insulin release observed in streptozocin-induced diabetic rats, and this has been related to the content in α-linolenic acid [75].

A tetracyclic triterpenoid, 16-hydroxy-4,4,10,13-tetramethyl-17-(4-methyl-pentl)-hexadecahydro-cyclopenta[*a*]phenanthren-3-one, isolated from the aerial part of *O. tenuiflorum*, was able to decrease serum glucose levels, total cholesterol, triglycerides and HDL cholesterol and to increase serum LDL cholesterol level in alloxan-induced diabetic rats [76].

### 2.5. Panax Ginseng C.A. Meyer, Radix and Panax quinquefolius L., Radix

Ginseng has been used in traditional Chinese medicine for more than 5000 years [77]. Thanks to its restorative, tonic, nootropic, and anti-aging properties [78], ginseng preparations have been applied to several pathological conditions such as hypodynamia, anorexia, shortness of breath, palpitation, insomnia, impotence, hemorrhage and diabetes [79].

Confirming the clinical strength of many of the traditional uses of ginseng, in 2014 the European Medicines Agency released a monograph which validated the use of ginseng as a traditional medicine for treating asthenia in Western countries too [80].

In medicine, the term ginseng may usually refers to several *Panax* species, which are often used indiscriminately. Nevertheless, the most commonly used are *Panax ginseng* C.A. Meyer and *Panax quinquefolius* L. In this review, the antidiabetic properties of *P. ginseng*, *P. quinquefolius* and their main constituents will be discussed together.

*Panax ginseng* C.A. Meyer is a perennial herb, native to Korea, Cina and Japan, with characteristic branched roots belonging to the family of Araliaceae. The herbal substance is represented by the dried roots, which main constituent are triterpenes saponins, known as ginsenosedes [19].

*Panax quinquefolius* L. is a perennial herb that is native to North America. The parts of the plant used are the dried roots, containing ginsenosedes, with a higher concentration of protopanaxadiols compared to *P. ginseng* [81].

Several systematic reviews described the potential of ginseng in the management of diabetes. In 2011, Kim and coworkers analyzed data deriving from four different randomized clinical trials in which ginseng (0.78–6 g/day for a maximum of 12 weeks) demonstrated no significant effects in controlling blood glucose in type 2 diabetes patients [82]. Nevertheless, promising results in improving glucose metabolism were found by Shergis and colleagues in 2013, by analyzing six clinical trials [83]. More recently, Shishtar and colleagues evaluated sixteen trials in which 0.1–20 g/day of different ginseng preparations were administered for 4–24 weeks to patients with and without diabetes. A modest yet significant reduction of fasting blood glucose was observed in both groups [84]. Finally, in 2016, a meta-analysis from Gui and coworkers failed to attribute significant improvement in hemoglobin A1c levels for ginseng treated patients (0.96–13.6 g/day for 4–20 weeks) alone and in combination with conventional therapies, compared to control group. However, improved fasting glucose and post prandial insulin levels were observed when ginseng was administered alone [85].

The effects of ginseng extracts and their main constituents have been extensively studied in vivo. Ginseng total saponins (150–300 mg/kg) significantly reduced hyperglycemia by increasing glucagone-like peptide-1 in high fat and low streptozocin-induced diabetic rats [86]. A fractionated extract containing water soluble ginseng polysaccharides, administered 1 g/kg/day for 2 weeks to streptozocin-induced diabetic rats, caused significant effects on purine, tryptophan, fatty acids and energy metabolism [87]. Protopanaxadiol and protopanaxatriol-type saponins (50–150 mg/day) reduced fasting blood glucose, glucose tolerance and insulin resistance in high fat and low streptozocin-induced diabetic rats. Among the mechanism of action analyzed, a suppression of TNF-α and IL-6 release, an increase in superoxide dismutase and a decrease in malondialdehyde levels, together with the downregulation of PPAR-γ coactivator 1α, phosphoenolpyruvate carboxykinase and glucose-6-phosphatase were observed [88]. In the same animal model, compound K, a metabolite of panaxadiol, (30–100–300 mg/kg) caused a dose dependent reduction of fasting blood glucose and an enhancement of fasting serum insulin and insulin sensitivity, by promoting the expression of insulin receptor, insulin receptor substrate-1, PI3Kp85, pAkt and GLUT-4 [89]. Ginsenoside-Rg1 was administered 10 mg/kg to streptozocin-induced diabetic mice, improving angiogenesis by increasing eNOS activation, VEGF expression and inhibiting apoptosis [90]. Two in vivo studies described the effects of 20(S)-ginsenoside-Rg3 (20 mg/kg) in streptozocin-induced diabetic rats. The first one reported a decrease in water intake and urine volume, together with a reduction in serum glucose, glycosylated protein and thiobarbituric acid-reactive substances production, leading to an improvement in renal dysfunction compared to control, which was related by the authors to the inhibition of NMDA receptor-mediated nitrosative stress [91]. The second further describes its positive effects on the metabolism of nucleic acid, energy and gut flora [92]. Finally, ginsenoside-Rh2 (1 mg/kg) lowered plasma glucose in streptozocin-induced diabetic rats, by increasing β-endorphin secretion, which is responsible for opioid μ-receptor activation, resulting in an increase of GLUT-4 expression [93].

It is then worth mentioning that several innovative extraction methods are currently under investigation in order to improve ginseng pharmaceutical applications. A heat-processed Korean ginseng extract was administered 100 mg/kg to streptozocin-induced diabetic rats, causing a reduction of blood glucose levels and an improvement of renal dysfunction without altering the expression of proteins involved in oxidative process, in a more potent manner compared to standard Korean ginseng extract [94]. Black ginseng, obtained by nine cycles of steaming and drying of ginseng, was administered 200 mg/kg to streptozocin-induced diabetic rats in two different studies. A modulation of glucose metabolism [95] as well as a more effective reduction of hyperglycemia, increase in insulin/glucose ratio and improvement of islet architecture and β-cells function was observed compared to standard red ginseng [96]. The inhibition of β-cells apoptosis was presumed by the authors to be related to the suppression of cytokine-induced NF-κB translocation. In the same animal model, a pectin lyase-modified ginseng extract (20–50–100 mg/kg) decreased the serum levels of AGE and their cross-linking with protein [97]. Finally, a tissue cultured mountain ginseng adventitious root extract enriched with ginsenosides, administered 250–500 mg/kg, was more effective than field cultivated Korean ginseng in lowering blood glucose, total cholesterol and triglycerides in streptozocin-induced diabetic rats [98].

### 2.6. Rehmannia glutinosa (Gaertn.) DC., Radix

*Rehmannia glutinosa* (Gaertn.) DC. is a perennial herb belonging to the family Plantaginaceae. The parts of the plant used are the dried roots and rhizomas [37]. The main constituents are iridoid glycosides and monoterpenes [99].

Within the various *R. glutinosa* preparations, the aqueous extract is preferred since it contains the largest amount of characteristic constituents [100]. The whole extract, the polysaccharides fraction and some isolated compounds exhibited beneficial activities in improving glucolipid metabolism and redox homeostasis (which is referred to as the balance between oxidant and antioxidant signaling [101]) in both in vitro and in vivo models. In particular, a water extract obtained from fresh rhizome exerted a high free radical scavenging activity and, in addition, was able to reduce ROS production, to suppress NF-κB activity and to down-regulate the expression of pro-inflammatory genes, such as TNF-α, COX-2, monocyte chemotactic protein-1 (MCP-1) and inducible protein-10 [102]. Furthermore, two different polysaccharide fractions exerted a significant hypoglycemic effect in normal, glucose- and alloxan-induced diabetic rats at 100 mg/kg (reducing hepatic glucose-6-phosphatase activity, increasing hepatic glycogen content, and raising plasma insulin levels) [103], and in streptozocin-induced diabetic mice at 20, 40 and 80 mg/kg (increasing the mRNA expression of phosphoenolpyruvate and the hepatic glycogen content) [104].

A water extract fraction containing approximately 60.51% of stachyose was administered 200 mg/kg/day for 15 days to normal, glucose-, adrenaline- and alloxan-induced diabetic rats, resulting in a significant hypoglycemic effect, although the mechanism of action was not investigated [105].

Catalpol at micromolar concentrations reduced AGE-induced inflammatory responses, inhibited the formation of intracellular ROS production and had a suppressive effect on NADPH-oxidase activity in THP-1 cells [106]. The protective effects of catalpol (20–120 mg/kg/day) through suppression of AGE-mediated inflammation have been extensively confirmed in animal models [107,108,109,110].

### 2.7. Trigonella foenum-graecum L., Semen

*Trigonella foenum-graecum* L., fenugreek, is an annual aromatic herb belonging to the family Fabaceae. The parts of the plant used are the dried ripe seeds, which contains mucilage and a variety of other secondary metabolites such as trigonelline [37].

A broad range of therapeutic uses, such as to ease childbirth, to increase milk flow, to alleviate menstrual pains and to treat body weakness, have been described in the traditional medicine of eastern Mediterranean areas [111].

Although results from clinical trials are substantially heterogeneous and there is still very limited evidence as to the impact of dietary consumption or supplementation with *T. foenum-graecum* for the management of diabetes, recent systematic reviews and meta-analysis conclude that fenugreek seeds (5–100 g/day) may be a promising complementary option for the clinical management of diabetes. This drug, indeed, contributes to a better glycemic control in type 2 diabetes mellitus patients, reducing fasting blood glucose, 2 h post load blood glucose and glycated haemoglobin [112,113,114,115].

In vivo studies agree on fenugreek’s hypoglycemic and hypolipemic activities: the seeds powder (5% in the diet for 21 days) decreased the level of lipid peroxidation in alloxan-induced diabetic rats [116], and significantly restored to control values the elevated fasting blood glucose levels in the same animal model (2 g/kg for 7 days) [117].

The mechanisms underlying fenugreek antidiabetic action include the lowering of blood glucose through an insulin signal pathway and the stimulation of glucose uptake in peripheral tissues [118].

The bioactive compounds which has been more deeply studied for the hypoglycemic actions are trigonelline, diosgenin, 4-hydroxyisoleucine and the soluble dietary fiber fraction of fenugreek seeds [119]. Aside from possessing antioxidant activity, trigonelline affects the activity of enzyme related to glucose metabolism, β-cell regeneration and insulin secretion [120]. Diosgenin is implicated in the renewal of pancreatic β-cells and in the stimulation of insulin secretion, has antioxidant effects and promotes adipocyte differentiation and enhancement of insulin-dependent glucose uptake [121,122,123]. 4-Hydroxyisoleucine stimulates glucose-dependent insulin secretion, reduces insulin resistance and inhibits sucrose α-d-glucosidase and α-amylase [124,125,126,127]. The soluble dietary fiber fraction of fenugreek (i.e., galactomannan) enhances glycemic control inhibiting lipid-hydrolyzing and carbohydrate-hydrolyzing enzymes in the digestive system and reducing the rate of glucose uptake [128,129,130].

## 3. Other Species with Promising Data for the Management of Diabetes

Several other species have been used in the ethnobotanical traditions of many countries around the world to treat diabetes [131,132,133], and most of them are under investigation for their potential role in the management of hyperglycemia and related diseases [134,135,136]. Currently, as retrieved by using the keywords “diabetes” and “phytotherapy” on PubMed, the clinical studies published on this topic are 254, and the review articles are 400 [137]. Nevertheless, the information on medicinal plants are various, with most of the species recording very few articles: many species are rarely used in Western medicine, but very common in other traditional medicine, such as traditional Chinese medicine [138]. Among the most used worldwide, the species reported in Section 2.1, Section 2.2, Section 2.3, Section 2.4, Section 2.5, Section 2.6 and Section 2.7 are the most studied. However, many other species have been recently considered by the scientific community.

*Gymnema sylvestre* (Retz.) R.Br. ex Sm., commonly known as gurmar, has been used since ancient times, particularly in Ayurvedic medicine, and its anti-obesity and anti-diabetic efficacy has been clinically demonstrated [139] and confirmed in animal models [140]. The anti-diabetic activity of gurmar has been attributed mainly to gymnemic acids, gymnemasaponins and gurmarin contained in the leaves [141]. A dihydroxygymnemic acetate (20 mg/kg), isolated from *G. sylvestre* leaves, has been administered in streptozocin-induced diabetic rats for 45 days, causing a significative reduction of plasma glucose and glycated hemoglobin level, and an increase of plasma insulin and muscle and liver glycogen [142]. The proposed mechanisms of action include the increase of insulin secretion and the promotion of islet cell regeneration, together with the reduction of intestinal and blood glucose adsorption [13]. Some products derived from *G. sylvestre* have been patented in different European countries. In 2010, a high molecular weight leaves extract (1 g/day for 60 days) was found to significantly increase the circulating insulin and C-peptide levels and reduced fasting and post-prandial blood glucose in a small cohort of patients with type 2 diabetes [143]. In the same year, a different *G. sylvestre* leaves extract (500 mg/day for 3 months) was similarly able to reduce fasting and post prandial blood glucose and glycated hemoglobin, causing a favourable shift in lipid profile, in type 2 diabetic patieints [144]. Nevertheless, more studies are needed in order to support the use of gurmar in the treatment of diabetic patients [145].

According to a recent systematic review, *Curcuma longa* L. can be considered a promising species for the management of impaired glucose tolerance, as curcumin is able to significantly reduce fasting blood glucose, glycosylated hemoglobin and insulin resistance after 3, 6 and 9 months of treatment [146]. Moreover, when administered together with an absorption enhancer (i.e., piperine 10 mg/day), curcuminoids (1000 mg/day) were able to reduce serum levels of atherogenic lipid indices, which are risk factors for cardiovascular events in type 2 diabetes patients [146].

*Morus alba* L. leaves demonstrated to possess a wide range of pharmacological activities in both in vitro and in vivo tests, including antidiabetic activity [147]. In a randomized double-blind placebo-controlled trial, the administration of *M. alba* leaf aqueous extract (5 g/day for 4 weeks) improved the postprandial glycemic control in patients with impaired fasting glucose tolerance, by reducing plasma glucose, insulin and C-peptide levels, compared to placebo [148].

The potential of ω-3 fatty acids on glycemic control is widely discussed [149]. *Linum usitatissimum* L. seed oil, also referred to as flaxseed oil, is contains over than 50% ω-3 fatty acids, mainly represented by α-linolenic acid [150]. A recent randomized controlled interventional trial demonstrated the ability of *L. usitatissimum* seed oil (25 mg/day) to ameliorate some symptoms of metabolic syndrome, including blood pressure and lipid peroxidation [151]. In type 2 diabetic patients with coronary heart disease, the supplementation with *L. usitatissimum* oil (1000 mg/day for 12 weeks) increased gene expression levels of PPAR-γ and downregulated gene expression of lipoprotein(a), IL-1 and TNF-α [152]. Moreover, *L. usitatissimum* seed powder (10 g/day for 1 month), containing not quantified ω-3 fatty acids and lignans, reduced fasting blood glucose, glycated hemoglobin, triglycerides, total and LDL cholesterol and apolipoprotein B, and increase HDL cholesterol levels in type 2 diabetes patients [153]. Similar results were obtained by the supplementation with *L. usitatissimum* gum (5 g/day for 3 months) [154].

A standardized extract of *Aristotelia chilensis* (Molina) Stuntz berries, containing ≥25% delphinidins and ≥35% anthocyanins, has been investigated in a double-blind placebo-controlled crossover trial in patients with impaired glucose regulation, showing a significant reduction of postprandial blood glucose levels, which was related to the inhibition of sodium glucose cotransporter (SGLT-1) [155]. However, in a three months clinical trial on prediabetic individuals, the same extract (180 mg/day) caused a reduction in glycated hemoglobin level, but no significant effects were reported for fasting insulin and glucose levels [156].

Insulin-like proteins from *Moringa oleifera* Lam. leaves have been addressed for their potential role in the management of diabetes [157]. In alloxan-induced diabetic mice, a leaf protein isolate from *M. oleifera* (500 mg/kg) reduced blood glucose level and oxidative stress, but did not stimulate insulin secretion [158]. A reduction of blood glucose and a high antioxidant activity was also observed in streptozocin-induced diabetic rats treated with a *M. oleifera* leaves methanol extract (250 mg/kg) [159]. However, clinical validation has to be carried out, since very little information is currently available, with only one preliminary clinical trial been conducted by administering *M. oleifera* leaf powder to healthy subject and evaluating outcome related to diabetes [160].

*Zingiber officinale* Roscoe has demonstrated beneficial effects on hyperglicemia in several experimental studies, by increasing insulin sensitivity and synthesis, and glucose uptake by tissues, and by reducing oxidative stress, whilst protecting pancreatic β-cells [161]. In a double-blind placebo-controlled randomized clinical trial, the administration of *Z. officinale* powder (3 g/day for months), containing not quantified gingerols, in type 2 diabetic patients reduced serum glucose and glycated hemoglobin levels, decreased insulin resistance and increase total antioxidant capacity [162].

The anti-diabetic potential of several *Morinda citrifolia* L. preparations has been reviewed recently: although there is a wide number of products on the market, there is still the need for well-conducted clinical trials in order to better investigate the role of this herbal product in diabetes [163].

The consumption of tea (*Camellia sinensis* (L.) Kuntze) and coffee (*Coffea Arabica* L.) has been related to a lowered risk of type 2 diabetes onset [164] and these beverage were also found to be effective in impaired glucose tolerance. Some of the effects of coffee involved in the prevention of type 2 diabetes includes: improvement of glucose tolerance, insulin sensitivity and insulin secretion, reduction of glucose intestinal uptake and regulation of glucose metabolism [165]. Moreover, a randomized acute crossover intervention study conducted in healthy volunteers showed the ability of coffee polyphenols to improve postprandial hyperglycemia, increasing glucagon-like peptide 1 secretion and decreasing oxidative stress [166]. Type 2 diabetic patients drinking three cups (600 mL) of black tea per day for 12 weeks reported a significant reduction in glycated hemoglobin level, together with an amelioration of the immune function relevant to prevention and management of type 2 diabetes [167]. The effect of tea seems to be related to its polyphenol contents, and in particular to epigallocatechin-3-gallate, which may act on glucose intestinal and cellular uptake and on oxidative stress [168].

Within the most studied hypoglycemic herbal products, cinnamon is undoubtedly attracting a great interest by the scientific community. For this reason, although there is a lack in authoritative documents reporting specific indications in diabetes, a section of this review is dedicated to a critical analysis of the available literature on cinnamon and its role in diabetes.

### Cinnamon

Botanical preparation of cinnamon may results from the dried inner bark of the shoots grown on cut stock of *Cinnamomum verum* J. Presl. as well as from the trunk bark, freed or cork of *Cinnamomum cassia* (L.) J. Presl., both species belonging to the family Lauraceae [19]. The parts of the plant used are the dried bark, free from the outer cork, which contains mainly cinnamaldehyde, eugenol and coumarin in concentrations that can vary abundantly between the two species [169]. Indeed, *C. verum* essential oil contains about 50–63% cinnamaldehyde and only traces of coumarins; *C. cassia* essential oil, instead, contains up to 95% cinnamaldehyde and up to 1% coumarins, together with an higher content of benzaldehyde and methoxycinnamaldehyde, compared to *C. verum* [170].

Many systematic reviews evaluating cinnamon effectiveness on diabetic patients has been published in the last five years. The oral consumption or supplementation with cinnamon, usually in combination with standard hypoglycemic medications or other lifestyle therapies, has been associated to modest effects on fasting plasma glucose and hemoglobin A1c [171], and to a decrease in levels of triglycerides, total and LDL cholesterol and to an increase in HDL cholesterol [172]. Although being judged as promising by Akilen and colleagues [173], the use of cinnamon on glycemic control need to be investigated in better conduced clinical trials [174,175,176].

In vitro and in vivo evidences indicate that cinnamon may have benefits in improving insulin sensitivity and glycaemic control. Its hypoglycaemic activity may be attributed to multiple mechanisms of action, including the stimulation of insulin release and insulin receptor signalling, the activation and regulation of enzymes involved in carbohydrate metabolism, glycolysis and gluconeogenesis (i.e., inhibition of pancreatic and intestinal amylase and glucosidase and increased of glycogen synthesis in the liver), stimulation of cellular glucose uptake and glycogen content (i.e., increased glucose transporter-4 receptor synthesis) and increased expression of PPARs [177,178,179,180,181,182,183,184,185,186].

It has been suggested that cinnamon’s effects on blood glucose can be attributed to its active constituent, cinnamaldehyde. The insulinotropic effects of cinnamaldehyde have been preliminarily investigated and are thought indeed to be responsible for promoting insulin release, enhancing insulin sensitivity, increasing insulin disposal, and exerting activity in the regulation of protein-tyrosine phosphatase 1B and insulin receptor kinase [180,182,187]. It is important to consider that the quantity of active cinnamaldehyde may vary among species and formulations [188].

Even if cinnamaldehyde content is higher in *C. cassia*, compared to *C. verum*, it is difficult to state which of the two species is more effective in the management of diabetes. Moreover, the long-term consumption of coumarins have been demonstrated to cause hepatoxicity in human [189,190] and, in 2008, the European Food Safety Authority confirmed the previously calculated theoretical added maximum daily intake for coumarins to 0.1 mg/kg bw [191]. Considering the potential toxicity of coumarins in *C. cassia*, it can be speculated that *C. verum* may be safer for clinical application in chronic diseases requiring prolonged treatments, such as type 2 diabetes.

## 4. Conclusions

Traditional medicine and ethnobotany are an enormous source of information on safety and biological effects of herbal products and many of the medicinal plants currently used to treat hyperglycemia, indeed, derive from traditional use. Medicinal plants possessing anti-diabetes activities, which use has been officially recognized in one or more World regions and is supported by clinical evidence, are considered by WHO and enlisted in WHO monographs on medicinal plants.

This review highlights the diverse and interesting actions that are attributable to edible plants such as onion, suggesting that simple food preferences could actually help in preventing metabolic diseases.

Among all the medicinal plants reviewed, ginseng and fenugreek possess stronger clinical evidence, and their use is supported not only by WHO monographs, but also by the EMA, that has summarized scientific data on these species, their preparations and chemical constituents in officially published assessment reports [192,193]

Cinnamon anti-diabetic activity lacks of authoritative support, but many clinical trials have been conducted in the last five years, suggesting an increasing interest concerning its application in the management of diabetes.

The most common hypoglycemic mechanisms of action found for the reviewed medicinal plants and their constituents include the inhibition of α-glucosidase and of AGE formation, the increase of GLUT-4 and PPARs expression and the antioxidant activity (Table 2). Moreover, the use of herbal products often relies on the synergistic and multitarget effects of the phytocomplex, which may lead to a clinical effectiveness together with a lower incidence of adverse events [194].

The comprehensive overview of the dataset suggests that dietary natural products and phytotherapy have today an interesting role in controlling the normal to border-line glucidic levels and as an integrative therapy, but they could not be considered as alternative drugs to mono-molecular ones for type II diabetic patients. Well conducted clinical trials using modern standardized extracts are of primordial importance and it is necessary to better investigate correlations between hypoglycemic activity and chemical composition of herbal preparations, with the aim of optimizing extracts to better trigger specific pathways and, finally, to propose correct dosages to enhance safety and effectiveness. Furthermore, as many species which use in the management of diabetes is not enlisted in authoritative documents such as WHO monographs are demonstrating interesting anti-diabetic properties in vitro and in vivo, and clinical trials are being conducted to demonstrate their effectiveness in human patients, it would be of great interest to carry out comparisons between the well documented species, such as those reported in Section 2.1, Section 2.2, Section 2.3, Section 2.4, Section 2.5, Section 2.6 and Section 2.7, and the new candidate species, such as those reported in Section 3, under similar experimental and clinical conditions.

## Figures and Tables

**Table 1:** Species enlisted in WHO monographs with indication of use for diabetes.

Use Supported by Clinical Data	Use Described in Pharmacopoeias and in Traditional Systems of Medicine
*Ocimum tenuiflorum* L., folium *Trigonella foenum-graecum* L., semen	*Allium cepa* L., bulbus
	*Azadirachta indica* A. Juss., folium
	*Momordica charantia* L., fructus
	*Ocimum tenuiflorum* L., folium
	*Panax ginseng* C.A. Meyer, radix
	*Panax quinquefolius* L., radix
	*Rehmannia glutinosa* (Gaertn.) DC., radix

**Table 2:** Pharmacological activities of the main chemical constituents of hypoglycemic medicinal plants.

Herbal Species	Main Chemical Constituents	Pharmacological Activities
*Allium cepa* L.	Quercetin	Inhibition of α-glucosidase
	Rutin	Increase of GLUT-4 translocation and glucose uptake; stimulation of insulin action
	l-cysteine sulfoxides Allyl-propyl disulphide	Free radical scavenging; increase of SOD and catalase activity
*Azadirachta indica* A. Juss.	Azadirachtins	Inhibition of α-amylase and α-glucosidase
*Momordica charantia* L.	Cucurbitane triterpenoids	Reduction of blood glucose levels; modulation of insulin secretion; stimulation of GLUT-4 translocation; upregulation of insulin receptor substrate-1; increase of AMPK phosphorylation
	EMCD	Reduction of TNF-α, iNOS expression and NF-κB nuclear translocation
	Momordin	Induction of PPARγ mRNA expression
*Ocimum tenuiflorum* L.	Essential oil	Reduction of lipid peroxidation; stimulation of antioxidant enzymes; stimulation of insulin secretion; free radical scavenging activity
*Panax ginseng* C.A. Meyer, and *Panax quinquefolius* L.	Protopanaxidiols	Increase of glucagone-like peptide-1; reduction of TNF-α and IL-6 release; increase of superoxide dismutase activity; reduction of malondialdehyde activity; down-regulation of PPAR-γ coactivator 1α, phosphoenolpyruvate carboxykinase and glucose-6-phosphatase; increase of insulin receptor substrate-1, PI3Kp85, pAkt and GLUT-4 mRNA expression
	Ginsenoside-Rg1	Induction of eNOS and VEGF expression; inhibition of apoptosis
	20(S)-ginsenoside-Rg3	Inhibition of NMDA receptor-mediated nitrosative stress; stimulation of nucleic acid and energy metabolism; positive effect on gut flora
	Ginsenoside-Rh2	Increase β-endorphin secretion; up-regulation of GLUT-4 expression
*Rehmannia glutinosa* (Gaertn.)	Polysaccharides	Improvement of redox homeostasis; reduction of hepatic glucose-6-phosphatase activity; increase of hepatic glycogen level; reduction of ROS production; inhibition of NF-κB translocation; down-regulation of TNF-α, COX-2, MCP-1 and inducible protein-10
	Catalpol	Inhibition of intracellular ROS production; suppression of NADPH-oxidase activity
*Trigonella foenum-graecum* L.	Trigonelline	Anti-oxidant activity; modulation of glucose metabolism; induction of β-cells regeneration
	Diosgenin	Increase of insulin secretion; induction of β-cells regeneration; anti-oxidant activity; promotion of adipocyte differentiation; enhancement of insulin-dependent glucose uptake
	4-hydroxyisoleucine	Stimulation of glucose-dependent insulin secretion; reduction of insulin resistance; inhibition of sucrose α-d-glucosidase and α-amylase
	Fiber	Inhibition of lipid- and carbohydrate-hydrolyzing enzymes; reduction of glucose uptake
*Gymnema sylvestre* (Retz.) R.Br. ex Sm.	Gymnemic acids; gymnemasaponins gurmarin	Increase of insulin secretion; induction of β-cells regeneration; reduction of intestinal and blood glucose uptake
*Cinnamomum verum* J. Presl. and *Cinnamomum cassia* (L.) J. Presl.	Cinnamaldehyde	Insulino tropic effect; regulation of protein-tyrosine phosphatase 1B; regulation of insulin receptor kinase; modulation of carbohydrate metabolism; inhibition of pancreatic and intestinal amylase and glucosidase; stimulation of cellular glucose uptake; increase of GLUT-4 expression; increase of PPARs expression.
